# Weekly High Dose Versus Daily Low Dose Vitamin D3 in Treatment of Vitamin D3 Deficiency in Pregnancy: A Randomized Controlled Clinical Trial 

**Published:** 2018-09

**Authors:** Farahnaz Rostami, Lida Moghaddam-Banaem, Sedigheh Hantoushzadeh

**Affiliations:** 1 Department of Midwifery and Reproductive Health, Faculty of Medical Sciences, Tarbiat Modares University, Tehran, Iran; 2 Maternal, Fetal and Neonatal Research Center, Tehran University of Medical Sciences, Tehran, Iran

**Keywords:** Pregnancy, Vitamin D Deficiency, Treatment

## Abstract

**Objective:** To compare administration of weekly high dose versus daily low dose vitamin D_3_ in treatment of vitamin D_3_ deficiency in pregnancy.

**Materials and methods:** A randomized controlled clinical trial was performed between July 2016 until 2 July 2017 on 215 pregnant women with vitamin D_3_ deficiency (serum levels < 30 ng/ml) and gestational age less than 14 weeks. The participants were randomly assigned to 2 treatment groups of A: receiving 1000 unit vitamin D_3_ daily, and B: 50,000 units weekly for 10 weeks. At 24-28 weeks of gestation, serum levels of vitamin D_3_ were measured again. Data entry and statistical analysis were performed by SPSS software v. 20 and P value less than 0.05 was considered as statistically significant level.

**Results:** Primary mean serum vitamin D3 level in group A was: 17.3 ± 6.8 and in group B: 15.2 ± 7.3 ng/ml while mean serum vitamin D3 level after treatment in group A was significantly lower than group B (31.9 ± 118 B vs. 42.9 ± 15.5, p-value: < 0.001); both groups were successfully treated, no remarkable side effects were observed in either groups.

**Conclusion:** As both regimens treat vitamin D deficiency successfully and consuming weekly high dose vitamin D_3_ makes more acceptable serum levels for mothers with no apparent side effects weekly high dose vitamin D_3 _can be safely administered for vitamin D_3_ deficiency in pregnancy, if further studies show similar results.

## Introduction

Vitamin D is a fat-soluble vitamin and a steroid hormone that has effects on the placenta, fetus and mother. Vitamin D deficiency epidemic is seen worldwide and its prevalence varies from 18% to 84% depending on the place of residence, country, ethnicity, customs, clothing and food consumption (1-3)**. **Vitamin D deficiency is one of the most common disorders among mothers and children (4). Vitamin D deficiency during pregnancy is associated with multiple adverse health outcomes in mothers, including gestational diabetes (5), pre-eclampsia (6) and bacterial vaginosis (7), and in newborns and children i.e. wheezing, low bone mineral density, type-1 diabetes and eczema (8). Despite the improvements in early diagnosis of vitamin D deficiency and availability of supplements and treatment modalities in the past few decades, hypovitaminosis D is still considered as a major public health concern, which is associated with significant morbidity in many countries (9). Previous studies have shown that vitamin D deficiency is common in pregnancy (10, 11). The prevalence of vitamin D deficiency has been reported to be between 60-80% among certain high-risk groups of pregnant women, particularly those with Middle Eastern origin like Iranian women (12, 13). A number of studies have been conducted to estimate vitamin D deficiency in Iran and various estimates have been reported (14). Reports indicate that 86% of Iranian pregnant women and 75% of their newborns suffer from vitamin D deficiency (15). It was stated in another report that despite widespread use of supplements during pregnancy, nearly 78% of pregnant Iranian women still had vitamin D deficiency (11). The difference between these estimates is partly due to the use of different cut-off levels for vitamin D deficiency, including < 20, < 25, and < 35 ng/mL as definition of vitamin D deficiency, in different studies. These evidences indicate that vitamin D deficiency is common among Iranian pregnant women and thus most pregnant women are at risk of vitamin D deficiency consequences. 

Most prenatal vitamins supplements contain 1000 to 1200 international units (IU) of vitamin D per serving. For pregnant women with vitamin D deficiency, UP to Date editors agree with American College of Obstetricians and Gynecologists (ACOG) ACOG and the Endocrine Society that 1000 to 2000 IU of vitamin D daily is safe (16) and may be necessary to maintain a blood level of vitamin D > 30 ng/mL (17). The 2011 ACOG Committee Opinion, recommended routine supplementation with the present dose of Vitamin D in a prenatal vitamin capsule, until more evidence is gathered to support a different dose (16). The 2011 Endocrine Society guidelines recommended that 1500–2000 IU daily vitamin D is required to reach the serum level 25 Hydroxyvitamin (OH) D level ≥ 75 nmol/L (18). Treatment for the majority of women who are vitamin D deficient, includes treatment for 4–6 weeks, either with cholecalciferol 20,000 IU once a week or ergocalciferol 10,000 IU twice a week, followed by standard supplementation. For women who require short-term repletion, 20000 IU weekly appears to be an effective and safe treatment of vitamin D deficiency. A 1000 IU daily dose is likely to be appropriate to maintain subsequent completion. In adults, very high doses of vitamin D (300,000 – 500, 000 IU intramuscular [IM] bolus) may be associated with an increased risk of fractures and thus such high doses are not recommended in pregnancy. A study conducted in 2011 demonstrated that supplemental doses of 4000 IU cholecalciferol a day was safe in pregnant women and was the most effective dose compared to lower doses (19). The latest UP to Date site report also stated that there is still insufficient evidence on the treatment of vitamin D deficiency with a 50,000 weekly dose of vitamin D for 6-8 weeks in pregnancy (20).

Vitamin D_3_ supplementation has been recommended in all guidelines about vitamin D deficiency in pregnancy. However, there are few studies on treatment of vitamin D deficiency during pregnancy as well as the most appropriate treatment dose in pregnancy. Various vitamin D treatment regimens have been compared in studies. Treatment with daily 1,000 units is accepted by almost all scientific communities such as ACOG and RCOG. However, there are limited studies on the treatment with a dose of 50,000 units of vitamin D weekly and there is no single study to compare 1000 units daily with 50,000 units weekly. Thus the aim of the present study was to compare the efficacy of 1000 units daily with 50,000 units weekly vitamin D_3_ in vitamin D deficient pregnant women in order to recommend the best therapeutic approach for Iranian pregnant women with vitamin D deficiency.

## Materials and methods

**Figure 1:**
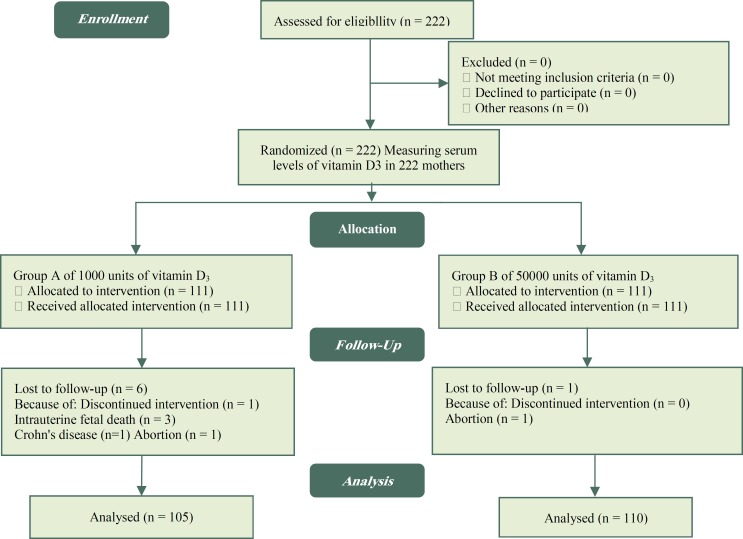
Consort flow chart

The present study was a randomized controlled and single-blinded clinical trial performed on 215 pregnant women with gestational age of less than 14 weeks. The age of mothers ranged between 18–42 years. Pregnant women and researchers were not blinded to treatment assignment but laboratory technicians were blinded regarding the type of treatment. Subjects were recruited from prenatal clinics in Tehran, Iran during July 2016-July 2017. The cut-off point for vitamin D deficiency was considered as serum levels of vitamin D less than 30 ng/ml. Two therapeutic doses of Vitamin D_3_ were administered to pregnant mothers who were diagnosed with vitamin D deficiency (25 (OH) D < 30 ng/ml). Vitamin D was measured by ELISA method using MAN Co kit. Inter assay and intra-assay coefficient of variations (CVs) were 1.9% and 1.1%, respectively. Baseline assessment of serum 25 (OH) D was performed in fasting condition along with the routine pregnancy tests in the first trimester of pregnancy. At first, a convenient sampling was performed and all pregnant women with vitamin D deficiency who were eligible were entered into the study(222 mothers) and then were divided into 2 groups of study (groups A and B, each comprising 111 subjects) based on random allocation software. From a total of 222 recruited subjects through random allocation sampling 7 participants were excluded because of crohn's disease (1), abortion (2), intra uterine fetal death (3) and discontinued intervention (1). Finally 215 pregnant women remained in study and were followed up every month during pregnancy and received vitamin D (Alhavi Pharmaceutical Co, Tehran, Iran) at doses of 1000 and 50,000 IU (Figure 1). In treatment Group A (105 subjects) mothers received 1000 IU vitamin D_3_ per day in the form of oral tablet from 14 to 28 weeks of gestation while Group B (110 subjects) were treated with 50,000 IU per week Vitamin D_3_ in the form of oral pearls for the same period of pregnancy. Gestational age was determined based on last menstrual period (LMP) for women with regular cycles and Ultrasound (model of GE LOGIC PRO 5) for those with irregular cycles at the beginning of the study. Evaluations included assessment of the adverse effects of vitamin D_3_, including headache and vomiting. Routine pregnancy tests were performed for all subjects in the second trimester. Consumption of other multivitamins supplementations were allowed during pregnancy. The data collection tools used in the study included a socio demographic questionnaire and anthropometric evaluations .Weight was measured with minimum clothing. Height was measured with a tape measure in standing position with normal posture of shoulders. Body Mass Index was calculated by dividing weight (kg) on height (m^2^). 

Systolic and diastolic blood pressures were measured twice in a sitting position with a standard mercury sphygmomanometer after a 15-min rest and the mean of the 2 measurements was considered as Systolic blood pressure (SBP) and Diastolic blood pressure (DBP). The study protocol was approved by the Medical Ethics Committee of the Research Tarbiat Modares University (IR.TMU.REC 1395.368). Participants were required to sign a written informed consent at recruitment covering all trial procedures and data collection. It was emphasized that participation in the study was voluntary and subjects were free to withdraw from the study at any time .Inclusion criteria comprised of gestational age < 14 weeks based on last menstrual period or ultrasound obstetrical estimation, age between 18 and 42 years, singleton pregnancy, lack of malabsorption or metabolic disorder, no history of consuming medications that affect appetite and absorption of nutrients and being of Iranian nationality. Subjects were excluded if they were unwilling to continue participation in the study, experienced any complications related to treatment (nausea, vomiting, fatigue, weakness, headache, frequent urination and dry mouth), incorrect use of administered vitamin supplement and being on any special diets, including vegetarian diet, etc.

To calculate the sample size, considering the lack of information about serum vitamin D_3_ levels in Iranian pregnant women, an experimental pilot study was conducted on 100 pregnant women in the first trimester of pregnancy and serum vitamin D_3_ levels were measured the obtained mean vitamin D_3_ level was 17.25 ng / ml with standard deviation of S = 15.8.

$n=\frac{{\left(\mathrm{Z1}-\frac{\propto }{2}+\mathrm{Z1}-B\right)}^{2}\times 2\times S^{2}}{{\left(∆\right)}^{2}}$

Average serum level of vitamin D_3 _in pilot study x = 17.25

20% minimum expected increase in serum vitamin D serum levels after treatment

S = 15/8, Z1- α / 2 = 96/1, Z1 -β = 8/0

n =$\frac{\left(96.1+0.84\right)²\times 2\times (8.15)²}{(3.45)²}$ = 87.5 

n = 87.5 _n _+10% possible drop out≅ 97

For each treatment group, there were about 100 people and a total of 200 participant. 222 participants were initially entered into the study.

 Continuous variables were checked for normal distribution using the one sample Kolmogorov-Smirnov test. Continuous data obtained from questionnaire and pregnancy tests were expressed as mean (standard deviation, SD) for normally distributed variables and median (interquartile range, IQR) for non-normally distributed variables while frequency (%) were used to describe categorical variables. Nonparametric tests were used in case of normality violation for continuous variables. Categorical variables were compared using chi-square tests. The Spearman correlation coefficient was used to assess the correlation between serum vitamin D_3_ levels before treatment with serum vitamin D_3_ level after treatment in both groups. Finally, Linear Regression model was used to evaluate the effectiveness of each treatment modality on increasing the serum levels of vitamin D_3_. Analyses was performed using the statistical package for social sciences (SPSS) software version 20 and p_value < 0.05 was considered as statistically significant level.

## Results

A total of 215 pregnant mothers with vitamin D_3_ deficiency completed the study in 2 groups (A: 105, B: 110). There were no significant differences between the 2 groups considering, demographic and reproductive characteristics at baseline (Table 1). 

**Table 1:** The baseline characteristics of the study participants (n = 215)

**Variable**	**Group B (50,000 units) ** **n = 11** **mean ± Standard deviation**	**Group A (1000 units) ** **n = 105** **mean ± Standard ** **deviation**	**P-** **value** *
Mother's age (years)	30.7 ± 5	31.7± 4.5	0.14
Gravid	2.17± 1.2	2.2± 1.1	0.30
Systolic blood pressure (mmHg)	106± 11.2	103± 11.4	0.56
Diastolic blood pressure (mmHg)	71± 11.5	70.6± 11.9	0.53
Gestational age based on ultrasonography in the first trimester (week)	11.7± 1.5	11.8± 1.5	0.96
BMI* of the first trimester of pregnancy (Kg/m^2)^	24.9± 4.08	24.9± 4.7	0.87
BMI pre-pregnancy (Kg/m^2)^	25.2± 4.2	25.2± 4.8	0.99

* T. Test

**Table 2:** Serum vitamin D level before and after treatment (n = 215)

**Variable**	**group A (1000 units)** **n = 105** **mean** ** ± ** **Standard ** **deviation**	**Group B (50,000units)** **n = 110** **mean ± Standard ** **deviation**	**P-** **value** *
Serum vitamin level D3 before treatment (ng / ml)	17.3 ± 6.8	15.2 ± 7.3	> 0.033
Serum vitamin level D3 after treatment (ng / ml)	31.9 ± 11.8	42.9 ± 15.5	>0.001
Increased serum vitamin D3 level after treatment (ng / ml**)**	27.76 ± 15	14.6 ± 9.6	< 0.001

* Mann-Whitney U Test

Statistically, with the Mann-Whitney U test, both groups were similar in terms of distribution of the above variables (age, number of pregnancies, blood pressure, gestational age and BMI).

Mann-Whitney U test showed that serum vitamin D_3_ level was significantly different between two groups before and after treatment. Before treatment, the 1000 unit and after the treatment group of 50,000 units show a higher level of vitamin D_3_ (Table 2).

The comparison of the difference between serum vitamin D levels before and after treatment showed that vitamin D serume levels after treatment increased with dose of 50,000 units, which was significant (Table 2).

In the follow up assessment with Chi-square test it was observed that in the treatment group, with 50,000 vitamin D3. Remaining vitamin D deficiency was much less than that of the 1,000 units. In general, in group B (50,000 units), vitamin D_3_ deficiency was untreated in less than one third of the subjects (13.6%). This finding indicates the lower failure rate of treatment less in group B (50,000 IU) (Table 3).

In the Linear Regression analysis, the dependent variable was the increase in serum vitamin D3 level after treatment. According to this analysis, despite taking other potentially effective vitamin D3 potentials considering other variables that may potentially affect treatment of vitamin D3 deficiency, only the type of treatment (treatment of 50,000 units) is effective in increasing VitD3 levels (Table 4).

## Discussion

In the present study, two doses for vitamin D_3_ administration were compared and the effect of both treatments was evaluated. Both treatments elevated serum vitamin D levels, despite lower levels of vitamin D_3_ in the 50,000 IU treatment group, indicating a greater effect for treatment at the dose of 50,000 IU in pregnant mothers. The acceptance of receiving oral treatment for vitamin D deficiency is high among pregnant mothers. Regardless of the importance of the necessity of vitamin D deficiency treatment in pregnancy due to its complications and reduced breast milk content of vitamin D, so far no study has been conducted to assess the effect of different doses of administration in pregnant mothers. In the present study, comparing different oral doses of vitamin D_3_ deficiency treatment in pregnancy was performed. In this non-randomized clinical trial we evaluated 215 pregnant mothers with serum vitamin D levels below 30 ng/ml. There was no significant difference in the duration of treatment between two groups. In case of vitamin D deficiency in pregnancy, most experts agree on the safety of administration of 1000–2000 IU per day. Higher dose regimens used for treatment of vitamin D deficiency have not been studied during pregnancy (16 .(Different studies suggested that the daily use 1000 IU(16, 21, 22), 2000 IU(23), 4000 IU(24) and 6400 IU(25) were safe. However, the effective dose of vitamin D to prevent or treat vitamin D deficiency in pregnancy is not clear. Some studies suggested that maintaining a blood concentration of vitamin D at above 50 nmol/l (20 ng/ml) requires the administration of around 1000 IU/d (26).

**Table 3:** The number remaining cases of vitamin D deficiency after treatment in mothers under study (n = 215)

**Variable**	**group A (1000 units)** **n = 105** **N(%)**	**Group B (50,000units)** **n = 110** **N(%)**	**P-value** *
Vitamin D3 deficiency (serum levels below 30 ng / ml)	55(52.4)	15(13.6)	>0.001

* Chi Square test

**Table 4:** The results of the evaluation of some of the variables that affect the elimination of vitamin D3 deficiency along with the type of treatment using Linear Regression model (n = 215)

**Effective factor**	**Beta ** **coefficient**	**P-** **value** *
Type of treatment	0.43	0.001
Age of mother	-0.003	0.66
Level of education ( up bachelor)	0.03	0.66
Job Status (employed)	0.044	0.51
Gravid	-0.041	0.62
Dose of vitamin D in multiprenatal	0.090	0.17
Gestational Diabetes	0.061	0.36
History of type 2 diabetes	-0.059	0.36
History of thyroid disease	0.033	0.52
BMI (before pregnancy)	0.040	0.60
Type of treatment	0.43	0.001

* Linear Regresion

Further research should focus on the potential benefits and optimal dosing of vitamin D in pregnancy. In this study, a significant increase in serum vitamin D levels was achieved in 105 pregnant mothers after 10 weeks of vitamin D_3_ administration of 1000 IU per day (mean increase in serum vitamin D levels was 31.9 ± 11.8 ng / ml). 

We used a 50,000 IU dose, which was shown to be safe in previous studies in treatment of vitamin D deficiency in rheumatology outpatient and health care professionals (27). Weekly doses of 50,000 IU vitamin D during pregnancy maintains acceptable vitamin D level during pregnancy and the vitamin D level in the newborns of these mothers correlates with the serum vitamin D levels of their mothers (28). Supplementation with < 50,000 IU/month is insufficient to ensure a vitamin D level > 20 ng/mL in all neonates born to vitamin D-deficient pregnant women (17). The therapeutic effect of deficiency and inadequate vitamin D_3_ in pregnancy on fetus growth and maternal weight gain in Iran in 2014 was assessed by Hashemi Pour et al (29). They administered 50,000 IU per week vitamin D_3_ to 65 pregnant women in the 26-24 weeks of gestation with vitamin D_3_ deficiency for up to 8 weeks until delivery, and observed improved growth rates in the embryo and increased maternal weight (30). In a randomised controlled trial of vitamin D during pregnancy, it was demonstrated that women with lower self-efficacy were more likely to experience practical problems with taking the trial medication, that was associated with lower compliance (30). In this study 10 week supplementation with 50,000 IU per week vitamin D_3_ in 110 pregnant mothers increased serum vitamin D_3_ levels to 42.96 ± 15.5 ng/ml, which was statistically significant and would help eliminate vitamin D insufficiency without apparent side effects. The strengths of the present study were to compare two therapeutic vitamin D doses (1000 with 50,000 units). So far, no study has been done in this regard. The study also had some limitations, including did not the inability to double blind two groups because of the specific treatment regimen.

## Conclusion

Despite the lower level of vitamin D_3_ in the 50,000 IU treatment group, vitamin D3 administration at this dose resulted in higher serum vitamin D levels after treatment compared to the 1000 IU group. The findings of this study provide evidence for the efficacy of administration of vitamin D_3_ at the dose of 50,000 IU per week for treatment of vitamin D deficiency in pregnancy. Furthermore, based on the findings of previous studies, and also this study, no prominent complications have been observed regarding the oral use of 50,000 IU per week vitamin D_3_, this dose might be safe in the treatment of vitamin D_3_ deficiency in pregnancy. Regarding the higher acceptability of weekly administration of vitamin D_3_ supplements, this treatment regimen might be more convenient for mothers to treat vitamin D_3_ deficiency during pregnancy. However, there is a need for larger studies with longer follow up periods in mothers and infants to ensure that no complications occur. It's better to study similar studies in other cities in Iran for vitamin D deficiency in pregnancy, to obtain a reliable and satisfactory result of the appropriate dosage for pregnancy.
